# Small Cell Neuroendocrine Carcinoma of the Bladder With Lymph Node Metastases: A Case Report

**DOI:** 10.7759/cureus.116353

**Published:** 2026-09-16

**Authors:** Souad El Ourdi, Othmane Zouiten, Leila Afani, Mohamed El Fadli, Rhizlane Belbaraka

**Affiliations:** 1 Department of Medical Oncology, Mohammed VI University Hospital Center of Marrakech, Marrakech, MAR

**Keywords:** bladder tumor, etoposide and cisplatin, neuroendocrine carcinoma of bladder, palliative cancer, rare cancers

## Abstract

Small cell neuroendocrine carcinoma (SCNEC) of the bladder accounts for less than one percent of all bladder cancers and is a highly aggressive and rare tumor. Rapid progression, early metastatic spread, and generally poor prognosis are the defining characteristics. Although there is currently no standardized therapeutic consensus, management is based on a multimodal approach that combines systemic chemotherapy, surgery, and/or radiotherapy.

In this report, we report the case of a 62-year-old female patient who presented with metastatic neuroendocrine bladder cancer involving the lymph nodes, evidenced by the presence of inguinal lymphadenopathy. To confirm the tumor's neuroendocrine nature, the patient underwent transurethral resection of the bladder tumor (TURBT) with placement of double-J ureteral stents. First-line chemotherapy with etoposide and carboplatin was subsequently initiated. The histological rarity of neuroendocrine bladder carcinoma underscores the uniqueness of this case.

## Introduction

Bladder cancer is a common malignancy of the urinary tract, with urothelial carcinoma accounting for more than 90% of cases. Non-urothelial epithelial malignancies are considerably less frequent and mainly include squamous cell carcinoma and adenocarcinoma. Neuroendocrine tumors of the urinary bladder represent a particularly rare group of neoplasms and include small cell neuroendocrine carcinoma (SCNEC), large cell neuroendocrine carcinoma, and mixed histological forms. Small cell carcinoma of the bladder (SCCB) accounts for approximately 0.5%-1% of all primary bladder malignancies and predominantly affects older men [1,2].

Although uncommon, these tumors are associated with poor prognosis and high metastatic potential. Their management is generally extrapolated from treatment strategies used for small cell lung cancer (SCLC), with platinum-based chemotherapy and etoposide forming the cornerstone of therapy.

A multimodal approach that includes radiotherapy and/or surgery may be taken into consideration for localized forms.

In this report, we describe the diagnostic workup and treatment of a patient with metastatic SCNEC of the bladder and discuss current management strategies based on the available literature.

## Case presentation

A 62-year-old female patient presented with total hematuria associated with progressive swelling of the right lower limb evolving over five months. Her medical history was notable for treated hypertension, with no significant previous urological history.

On admission, the patient had a moderate deterioration in her general condition, with asthenia. Physical examination revealed a firm, tender right inguinal lymph node measuring approximately 2 cm, while abdominal, urological, and gynecological examinations were otherwise unremarkable.

Thoraco-abdomino-pelvic CT revealed a 22-mm thickening of the posterior bladder wall involving the ureteral orifices, associated with multiple lymphadenopathies involving the interaortocaval, preaortic, para-aortic, right external iliac, and right inguinal regions, the largest measuring 25 × 14 mm. No visceral metastases were identified. The right external iliac lymph nodes were considered regional, whereas the para-aortic and inguinal lymphadenopathies represented non-regional nodal metastases (M1a) (Figure 1). Excision of an inguinal lymph node revealed a SCNEC.

**Figure 1:**
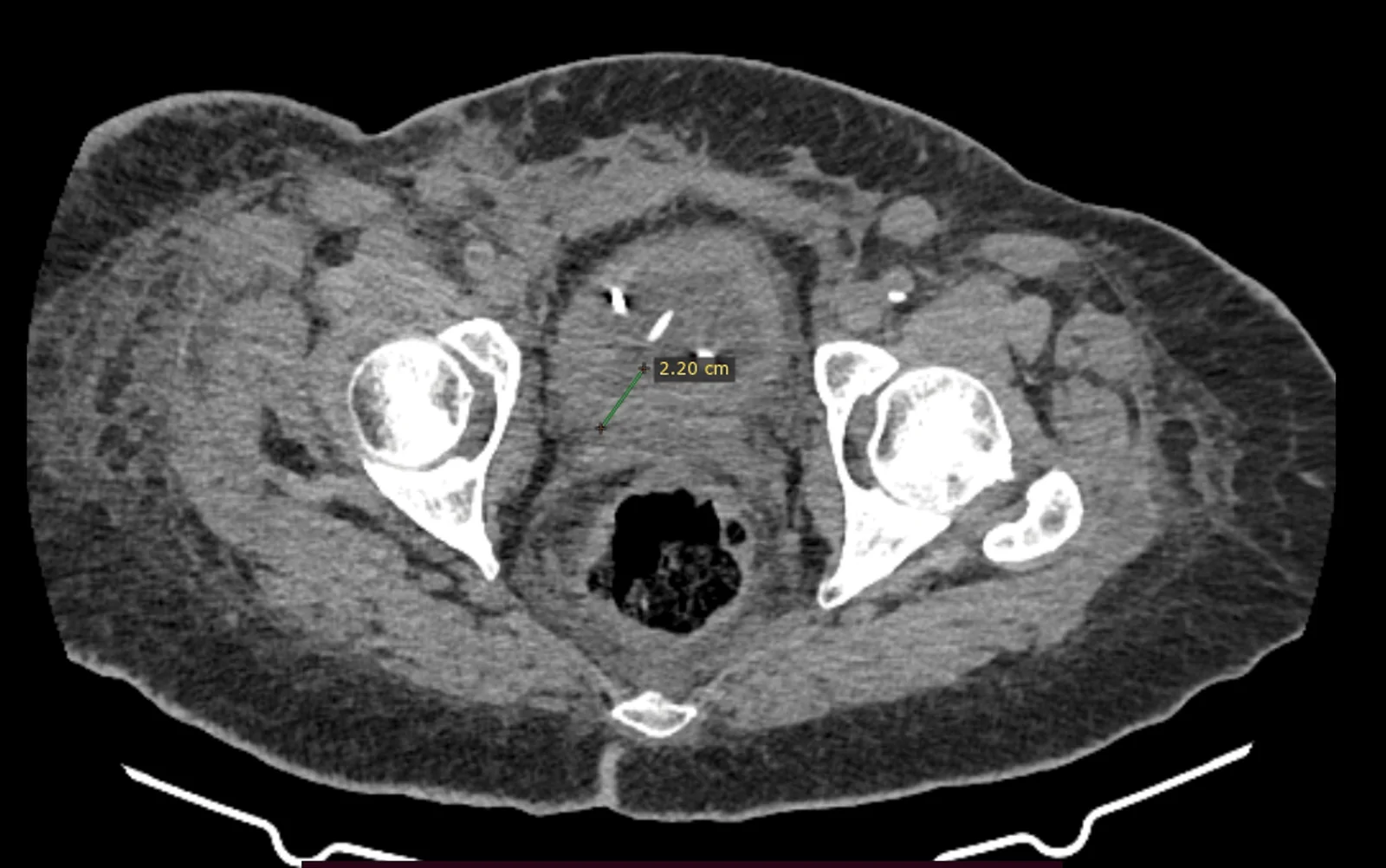
Thickening of the posterior wall of the bladder measuring 22 mm.

Immunohistochemical analysis showed positive expression of chromogranin and synaptophysin, with a CK7-/CK20+ immunoprofile and a Ki-67 proliferation index of 50%.

Molecular subtyping/next-generation sequencing (NGS) was not performed in our setting. This represents a limitation, as molecular profiling may refine prognosis and facilitate enrollment in clinical trials.

Following multidisciplinary discussion, the patient underwent transurethral resection of the bladder tumor (TURBT) for local tumor management, with bilateral double-J ureteral stent placement to relieve ureteral obstruction caused by tumor involvement of the ureteral orifices.

Histopathological examination revealed a tumor proliferation with a neuroendocrine architectural pattern, composed of small tumor cells infiltrating the detrusor muscle. Immunohistochemical analysis showed cytoplasmic positivity for the neuroendocrine markers synaptophysin and chromogranin, supporting neuroendocrine differentiation. The Ki-67 proliferation index was approximately 60%, consistent with a highly proliferative neuroendocrine carcinoma. Tumor cells showed a CK7-/CK20+ immunoprofile; however, this profile is not specific for a primary bladder origin and was therefore interpreted in conjunction with the histomorphological findings, neuroendocrine marker expression, and clinical and radiological assessment excluding another primary site. Overall, the morphological and immunohistochemical findings were consistent with SCNEC of the bladder infiltrating the detrusor muscle (pT2), with vascular emboli and perineural invasion (Figures 2-5).

**Figure 2:**
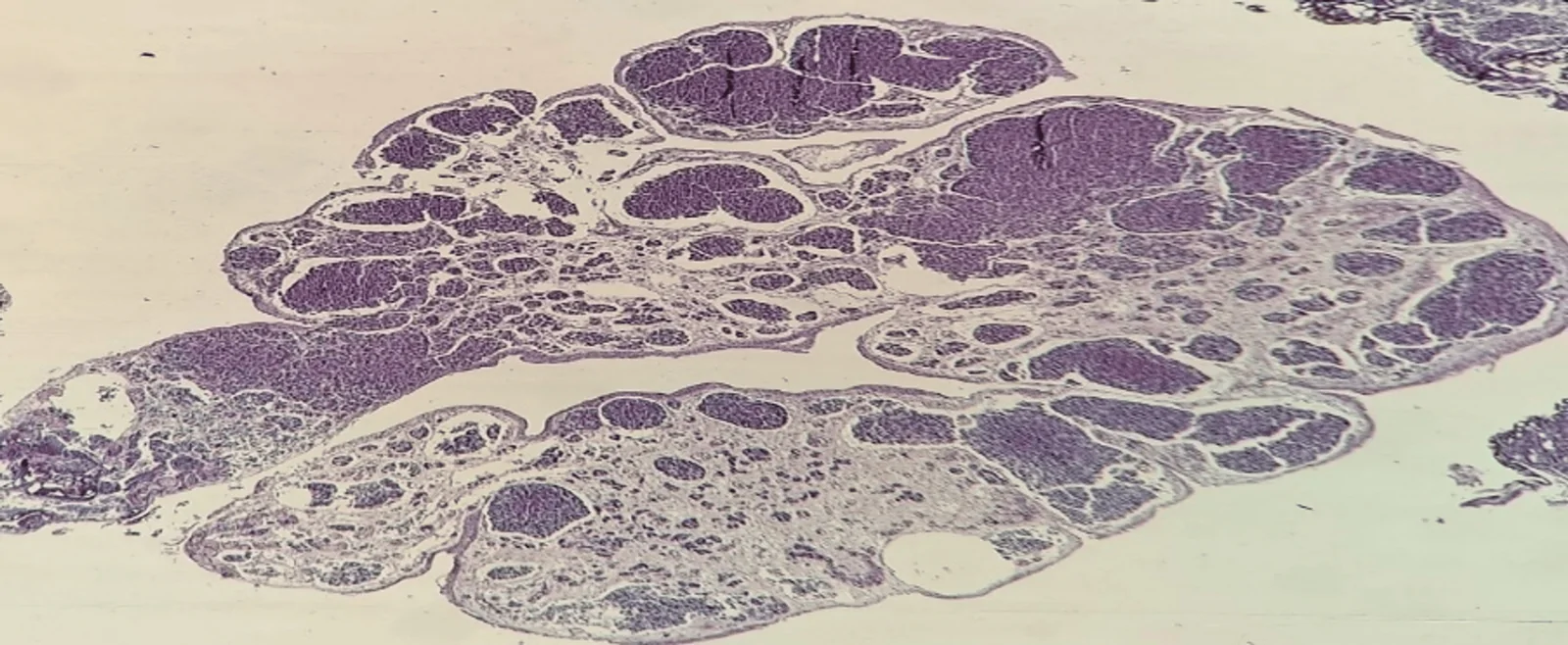
Low-magnification view of the urothelial mucosa with tumor proliferation arranged in lobules, using standard hematoxylin-eosin staining.

**Figure 3:**
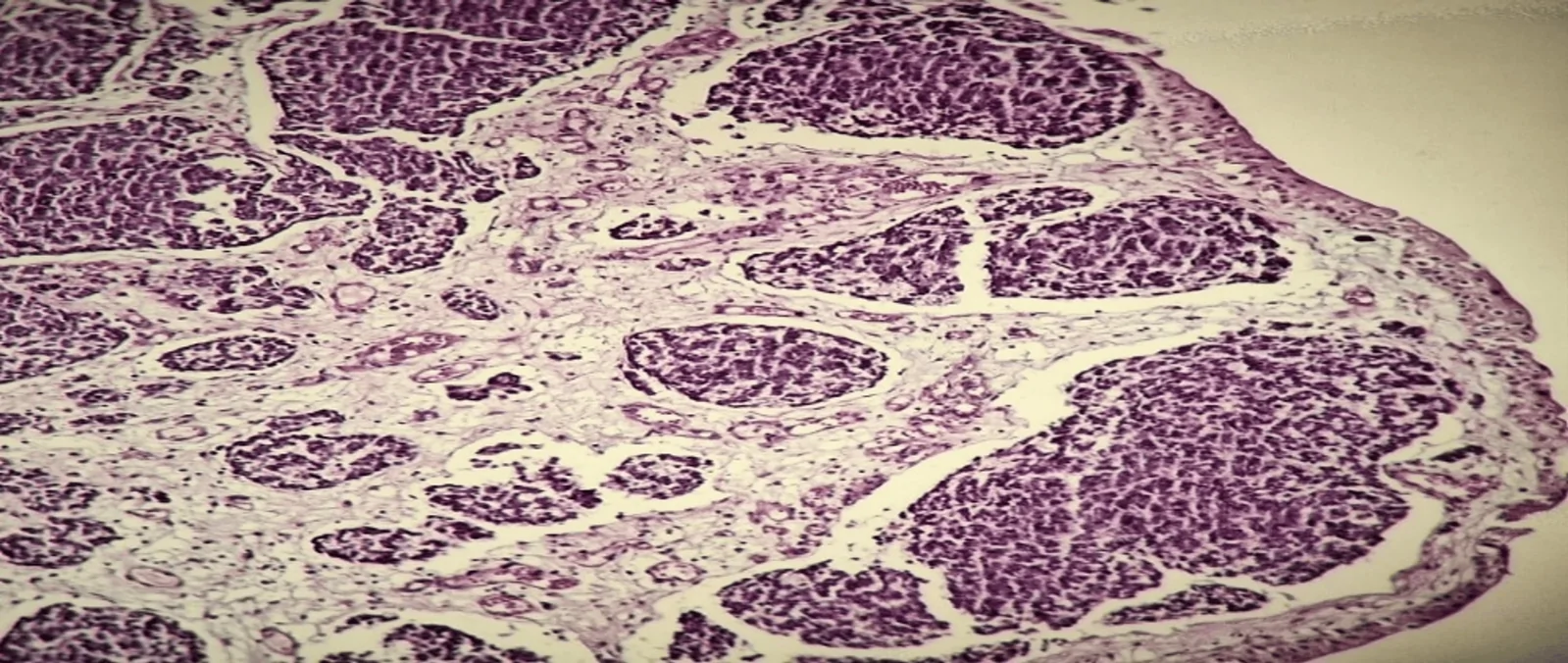
High-magnification view with standard H&E staining, showing small, hyperchromatic tumor cells with a high nucleus-to-cytoplasm ratio.

**Figure 4:**
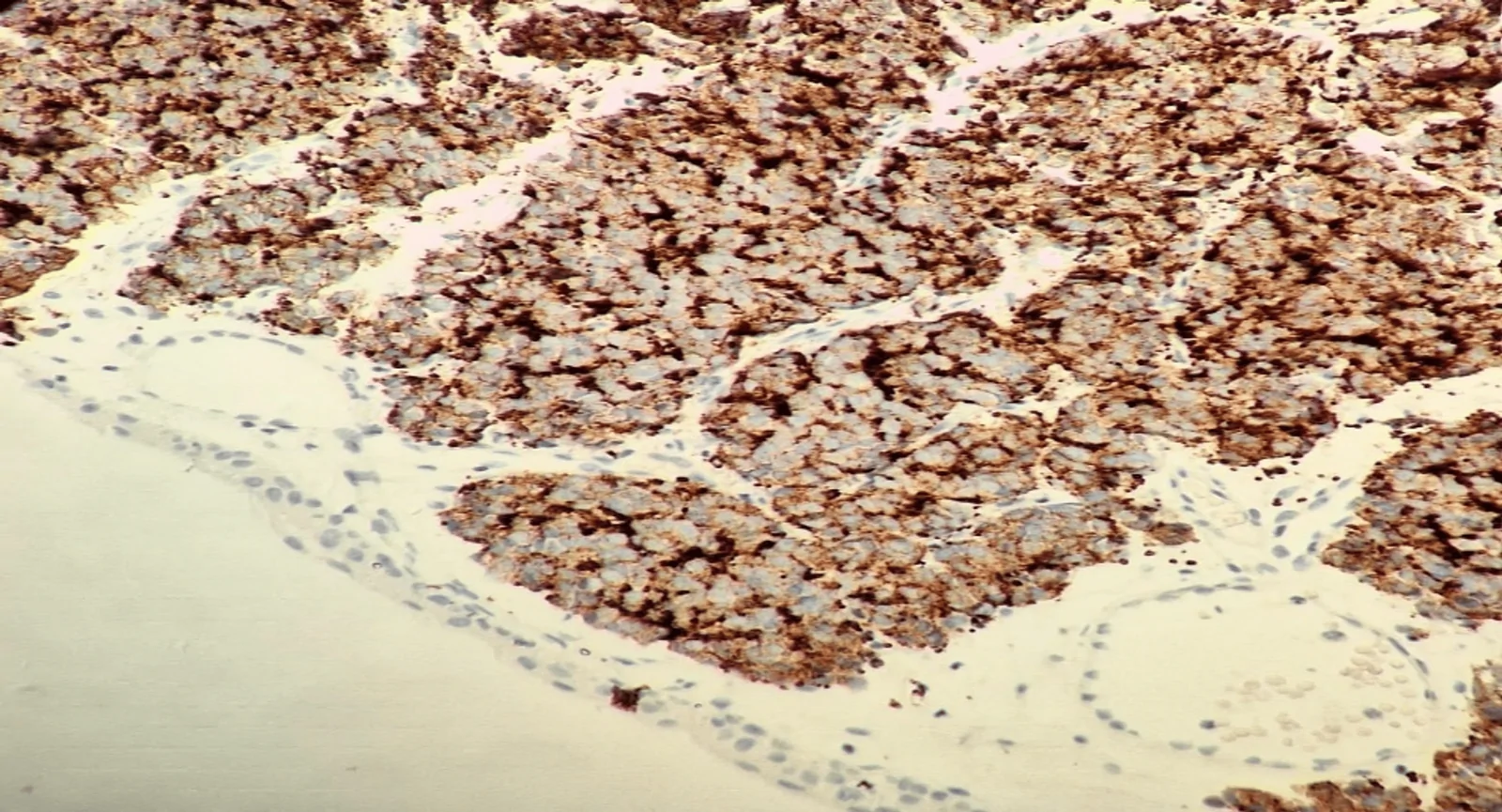
Immunohistochemical profile showing moderate to strong granular cytoplasmic staining of tumor cells with the anti-synaptophysin antibody.

**Figure 5:**
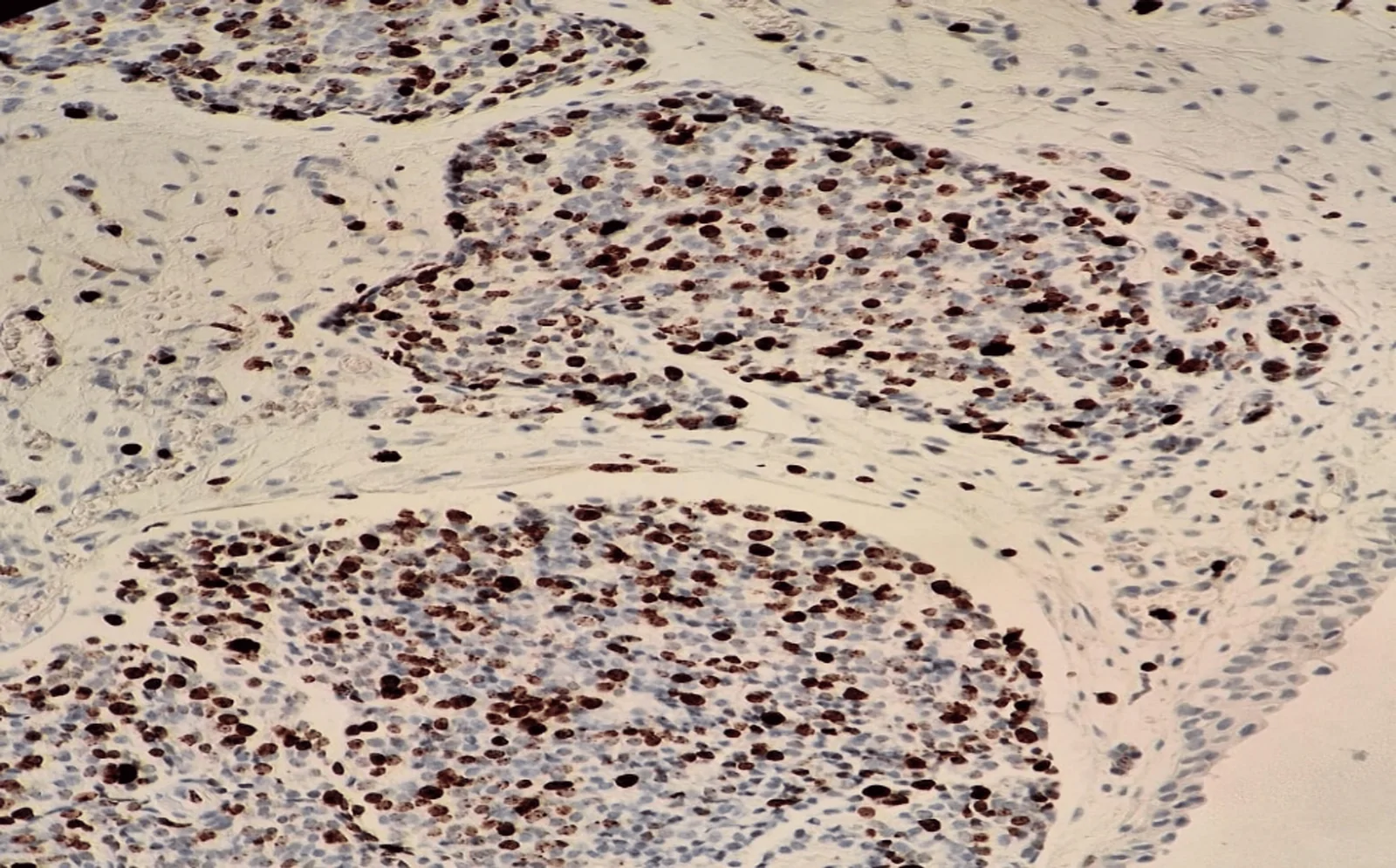
Moderate to intense nuclear expression of Ki-67 (proliferation index) in 60% of tumor cells.

Given the metastatic involvement of lymph nodes and the patient's ineligibility for cisplatin due to severe renal impairment (creatinine clearance of 28.7 mL/min), first-line chemotherapy was initiated with a combination of etoposide (100 mg/m² IV, days 1 to 3) and carboplatin (AUC 5, day 1).

The patient received three cycles of carboplatin and etoposide, with poor tolerance and progressive deterioration of her general condition between cycles. During treatment, she developed severe renal failure that subsequently progressed to end-stage renal disease. Given the marked deterioration in her general condition and renal function, further chemotherapy could not be administered. No radiological response assessment could be performed because of the rapid deterioration of her clinical condition. Despite supportive care, her clinical condition continued to worsen, and she subsequently died.

## Discussion

The bladder is a rare site of neuroendocrine carcinomas, first described by Cramer et al. in 1981 [1]. In contrast, the lung remains the most frequent primary location for these tumors.

In the present case, the absence of another identifiable primary malignancy on the staging workup, particularly in more common sites such as the lung, together with the presence of a bladder lesion, supported a primary bladder origin rather than metastatic neuroendocrine carcinoma from another site. Nevertheless, in the setting of metastatic disease, this distinction does not substantially alter the systemic therapeutic approach or the overall prognosis.

Neuroendocrine carcinomas account for approximately 0.5%-1% of all bladder malignancies [2]. They typically affect patients over the age of 50 and occur more commonly in men, with a male-to-female sex ratio of approximately 3.6. The most frequent initial symptom is hematuria [3], and unlike pulmonary forms, these tumors are typically not associated with paraneoplastic syndromes.

These tumors are aggressive in nature; metastases are found in 28% to 50% of cases, and over 70% of cases are found at an advanced stage. The brain, lungs, bones, liver, and lymph nodes are the organs most frequently affected by metastases. There is no established therapeutic consensus due to the rarity of this entity [4].

Palliative chemotherapy is the mainstay of treatment for metastatic forms. The most widely used protocols are based on cisplatin and etoposide [5], although other regimens that combine cisplatin and etoposide with doxorubicin-cyclophosphamide-vincristine or ifosfamide-doxorubicin have also been reported [6].

Recently, immunotherapy has emerged as a potential treatment option, showing encouraging but still limited results [6,7]. Wilde et al. reported a case demonstrating the efficacy of pembrolizumab in a patient who progressed after multiple lines of chemotherapy, achieving a durable and well-tolerated partial response [8]. Ongoing clinical studies, such as a phase Ib trial assessing pembrolizumab combined with platinum-based chemotherapy for neuroendocrine cancers of the prostate and urothelium [9], are being conducted to investigate the effectiveness of this combination.

Radiotherapy is mainly considered for symptom control and local palliation (hematuria or pain) and may be discussed as part of a multimodal strategy in selected cases.

The overall five-year survival rate for all forms is 16 to 25%, and the median survival is less than nine months. Overall, the prognosis is poor.

According to a recent systematic review and meta-analysis by the European Association of Urology, bladder small cell carcinoma has a significantly lower overall survival rate than urothelial carcinoma, making it an independent poor prognostic factor [10].

## Conclusions

Bladder SCNEC is a rare and highly aggressive malignancy with a high metastatic potential and poor prognosis. Diagnosis relies on histopathological and immunohistochemical findings. Because of the limited data available for this disease, treatment is generally based on experience with SCLC. Platinum-based chemotherapy is commonly used, with surgery or radiotherapy considered in selected cases, particularly for localized disease. Treatment decisions should be discussed within a multidisciplinary team and adapted to the clinical presentation and disease stage. Larger prospective studies are required to clarify the most effective management of this uncommon bladder tumor.
